# A Monster to Be Avoided

**Published:** 1891-09

**Authors:** C. B. Adams


### A MONSTER TO BE AVOIDED.

The monster that Columbus met and befriended in the American forests; that Hernandez introduced into Europe; that Raleigh brought to England; that James the First, and Pope Urban XIII., and the Russian government, and Ammat, the Sultan of Turkey, all warred with, Tobacco—monarch mightier than Alexander or Napoleon—yet robs our ranks. Young men are falling. The strength of the land, the "trustees of posterity," are failing us. Devotees may hug their idol and call us radically puritanic, but competent scientific men have carefully diagnosticated the demon weed. Of itself the fearful plant is deleterious enough, for its very essence—that which produces the soothing, fascinating, and enslaving effect—the nicotine—chemists and authoritative medical authors unanimously pronounce "in its action on the animal system one of the most virulent poisons known." A single drop put on the tongue of a cat produced violent convulsions, and killed her in the space of one minute; while a thread dipped in the same oil, and drawn through a wound made by a needle in an animal, killed it in the space of seven minutes.

More shocking, however, is the revelation concerning the constituents of the bane of boyhood, denominated cigarette. According to Professor Laflin, all of these "coffin nails" contain five distinct poisons. Three are the most deadly oils, one in the wrapper, one in the nicotine, and the third, the worst, in the flavoring. The other two are saltpetre and opium. When the smoker draws the smoke into his lungs, upon those vital organs and the throat is continually deposited a brown stain, a combined coating of all five poisons, which in time stains the very skin, thus indicating that the whole system is permeated by it.

Now, is it mysterious that the cigarette is so seductive, and obtains such fatal power, when in it is that pseudo-panacea of Turks and Chinese, which racked and ruined DeQuincey and Coleridge?

Again, as to cigarettes: In a single winter 52 young men died in Brooklyn, N. Y. A dozen boys became insane, and are confined in the Napa, Cal., hospital. A young fellow of sixteen dies in Philadelphia, and the post mortem shows death due to congestion of the brain. One, the brightest in his class, begins to smoke; a year later is stricken with heart disease—then dies in Louisville. In Kingston, N. Y., a young man suffers from the hallucination that he is followed by the police, who think him "Jack the Ripper," fled from London. Directly descended from three eminent ministers, and himself dedicated to missionary work, eighteen years old, a young man of fine promise, but now mentally diseased, wanders from home and friends. A girl, employed to fold the poisonous packages, dies as the result. Dr. Seaver, of Yale College, after an eight years' observation of the effects of tobacco smoking upon Yale students, comparing indulgers with abstainers, informs us that the former have less lung power, less chest inflating capacity, less bodily weight, and even less height. Also in an intellectual way, he says, Yale smokers are inferior to the anti-smokers. Of those students, who, within a given time, have received junior honors above "dissertations," only five per cent. were smokers, and very few smokers received appointments of any kind.

Notwithstanding all these frightful facts, the production proceeds, the consumption continues, and a multitude are being dwarfed in body, mind and soul.—C. B. ADAMS, *Episcopal Methodist*, Balt.

